# Germ cell depletion in recipient testis has adverse effects on spermatogenesis in orthotopically transplanted testis pieces via retinoic acid insufficiency

**DOI:** 10.1038/s41598-020-67595-1

**Published:** 2020-07-01

**Authors:** Akihiro Tsuchimoto, Masaaki Tone, Narumi Ogonuki, Masashi Hada, Atsuo Ogura, Seiji Takashima

**Affiliations:** 1 Department of Textile Science and Technology, Interdisciplinary Graduate School of Science and Technology, Shinshu University, Ueda, 386-8567 Japan; 2 Department of Applied Biology, Faculty of Textile Science and Technology, Shinshu University, Ueda, 386-8567 Japan; 3 Bioresource Engineering Division, Bioresource Research Center, RIKEN, Tsukuba, 305-0074 Japan; 4 Department of Biotechnology, Institute for Biomedical Sciences, Interdisciplinary Cluster for Cutting Edge Research, Shinshu University, Matsumoto, 390-8621 Japan

**Keywords:** Stem-cell niche, Stem-cell niche

## Abstract

Germ cell depletion in recipient testes is indispensable for successful transplantation of spermatogonial stem cells. However, we found that such treatment had an adverse effect on spermatogenesis of orthotopically transplanted donor testis tissues. In the donor tissue, the frequency of stimulated by retinoic acid (RA) 8 (STRA8) expression was reduced in germ cells, suggesting that RA signalling indispensable for spermatogenesis was attenuated in germ cell-depleted recipient testes. In this context, germ cell depletion diminished expression of testicular *Aldh1a2*, which is responsible for testicular RA synthesis, while *Cyp26b1*, which is responsible for testicular RA metabolism, was still expressed even after germ cell depletion, suggesting an alteration of the RA synthesis/metabolism ratio. These observations suggested that RA insufficiency was one of the causes of the defective donor spermatogenesis. Indeed, repetitive RA administrations significantly improved donor spermatogenesis to produce fertile offspring without any side effects. These findings may contribute to improving fertility preservation techniques for males, especially to prevent iatrogenic infertility induced by chemotherapy in prepubertal cancer patients.

## Introduction

Spermatogonial stem cells (SSCs), a subset of undifferentiated spermatogonia, maintain their population by glial cell line-derived neurotrophic factor (GDNF)-mediated self-renewal^1^. Undifferentiated spermatogonia are induced to differentiate into differentiating spermatogonia by retinoic acid (RA). This molecule also mediates the next differentiation step, namely differentiation of differentiating spermatogonia into spermatocytes^2,3^. These processes require support from the germline niche consisting of Sertoli and Leydig cells in addition to the basement membrane^4,5^. The former cell type was found by Enrico Sertoli as ‘nurse cells’ for germ cells, which produces GDNF and form a blood-testis barrier, both of which are indispensable for proper spermatogenesis^6^. The latter cell type found by Franz Leydig synthesises androgens depending on luteinising hormone to regulate Sertoli cell functions^7^. Recent reports have demonstrated that residential macrophages, peritubular myoid cells, and neighbouring lymphatic epithelial cells also have some effects on spermatogenic processes^8–11^. Moreover, the hypothalamic-pituitary axis further controls Leydig cell functions via luteinising hormones to produce testosterone^12^. Oestradiol synthesised from testosterone by aromatase in Leydig cells is also dispensable for proper spermatogenesis^13,14^.

Although spermatogenesis can be impaired because of several reasons, cancer treatments such as anti-cancer drugs, irradiation, and orchiectomy have a significant effect on the fertility prognosis^15^. In particular, although adult patients can avoid complete loss of fertility by freeze stocking of their own sperm before cancer therapy, this strategy is not applicable to prepubertal patients whose testes are too immature to produce haploid germ cells applicable to microinsemination^16,17^. Considering the recent strategic improvements in cancer therapy, cancer survivors suffering from male infertility will continue to increase.

Thus far, there are three techniques to restore spermatogenesis: spermatogonial transplantation, in vitro spermatogenesis, and testis piece transplantation. Spermatogonial transplantation is able to restore the fertility of infertile male mice^18^. This technique is applicable to transplantation of frozen and allogenic SSCs^19,20^. Although successful xenotransplantation of rat and hamster spermatogonia into infertile nude mice has been achieved for offspring production, offspring has not been obtained from dogs, rabbits, cattle, pigs, baboons, and humans even in allogenic transplantation^21^. In vitro spermatogenesis is a recently established technique to restore spermatogenesis. Sato and colleagues developed a testis culture system in which testis pieces from pup/adult mice are cultured on an agarose gel block for more efficient oxygen uptake by the tissues at the liquid–air interface^22,23^. However, this technique is not applicable to mammals other than mice^24^.

Based on these contexts, it seems that testis piece transplantation is the most practical technique to restore male fertility in mammalian species at present. Indeed, this technique has already been applied to not only functional analysis of mutant mouse testes, but also restoration of spermatogenesis in other mammalian species^25,26^. Moreover, successful offspring production has already been achieved in mice, rabbits, pigs, and primates by this technique^27–29^.

However, we found that germ cell depletion by the anti-cancer drug busulfan compromises the spermatogenesis of orthotopically transplanted donor testes, whereas testis pieces transplanted into healthy testes result in proper spermatogenesis. This observation is inconsistent with previous observations that germ cell depletion dramatically enhances colonisation, SSC-originating spermatogenesis, and offspring production^30,31^. Therefore, in the present study, we examined why depletion of recipient germ cells affects the spermatogenesis of orthotopically transplanted testis pieces, and found that recipient germ cell depletion caused RA insufficiency that compromised the spermatogenesis of orthotopically transplanted donor testis pieces.

## Results

### Germ cell depletion in recipient testes compromises spermatogenesis in donor testis pieces

**Figure 1:**
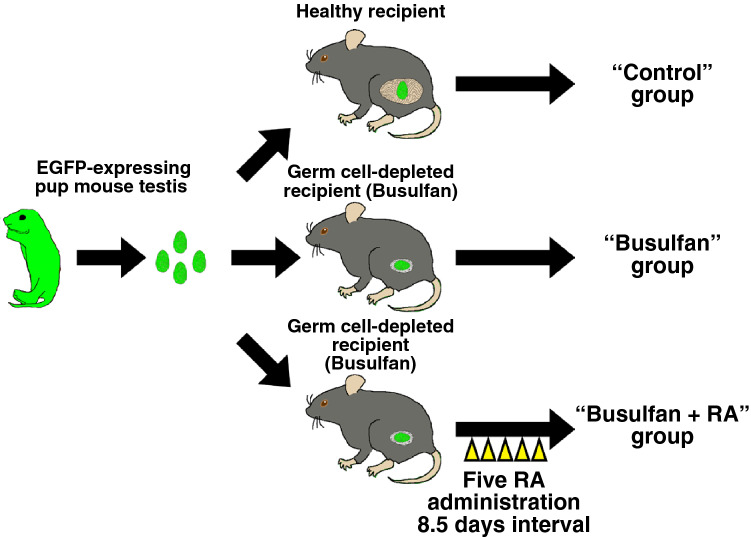
Experimental procedure. Testes were collected from neonatal mice constitutively expressing EGFP and transplanted into recipient testes. Untreated recipients were designated as the control group. Some recipients (busulfan group) were administrated with busulfan at a dose of 44 mg/kg b.w. that is sufficient to deplete germ cells completely. In addition, some germ cell-depleted recipients were also administrated retinoic acid at a dose of 750 µg/body five times with an 8.5-day interval (yellow arrowheads) just prior to sacrifice (busulfan + RA group). Transplanted testis pieces were collected after around 146 days (139–154 days) and subjected for immunofluorescence staining.

**Figure 2:**
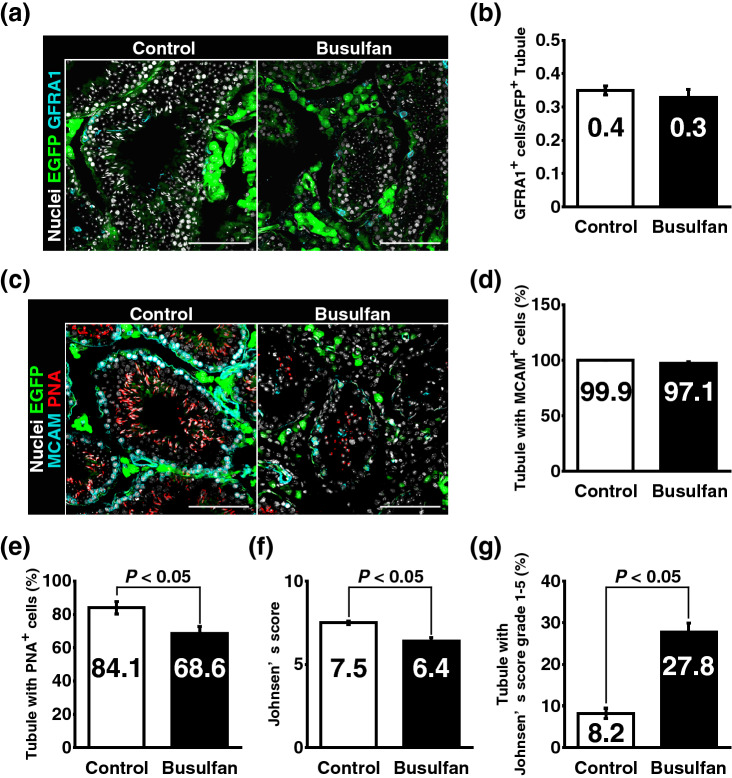
Recipient germ cell depletion compromises spermatogenesis in recipient testes transplanted into the testis interstitium. Testis pieces from green mouse pups were transplanted into the testis interstitium of control and busulfan groups. At around 146 days after transplantation, transplanted testis pieces were collected and subjected to immunofluorescence staining. (**a**) GFRA1 staining (cyan). Nuclei were counterstained with Hoechst 33342 (grey). EGFP expression (green) indicated that the tissues were derived from donor testes. (**b**) Quantitative analysis of GFRA1^+^ spermatogonia in transplanted testis pieces. Control group, n = 6 testes; busulfan group, n = 7 testes. A total of 4,366 tubules carrying 1,454 GFRA1^+^ spermatogonia were analysed. (**c**) MCAM (cyan) and PNA (red) staining. Acrosomes were labelled with PNA. EGFP expression indicated that the tissues were derived from transplanted testis pieces. Nuclei were counterstained with Hoechst 33342 (grey). (**d**,**e**) Quantitative analysis of MCAM^+^ germ cells and PNA^+^ haploid germ cells in transplanted testis pieces. Control group, n = 6 testes; busulfan group, n = 7 testes. A total of 4,007 tubules in which 3,934 tubules carried MCAM^+^ germ cells and 2,896 tubules carried PNA^+^ haploid germ cells were analysed. (**f**,**g**) Analysis of tubules by Johnsen’s score count. Tubules analysed in **d**,**e** were scored based on the criteria in a previous report^32^. Bar 100 μm (**a**,**c**).

Testis pieces collected from mouse pups expressing enhanced green fluorescent protein (EGFP) constitutively (green mice) were transplanted into the testicular interstitium of recipient testes with or without germ cell depletion induced by busulfan (Fig. 1). Although it has been demonstrated that germ cell-depleted testes are more suitable hosts for SSC transplantation than healthy testes with intact germ cells^27,28^, we found that the number of GFRA1^+^ undifferentiated spermatogonia and the frequency of seminiferous tubules carrying MCAM^+^ spermatogonia were comparable between testis pieces transplanted into busulfan-treated testes and those in control recipients (Fig. 2a–d, SI Fig. S1a–d). Moreover, we found that the frequency of seminiferous tubules containing PNA^+^ haploid germ cells was reduced significantly in testis pieces transplanted into busulfan-treated recipients (Fig. 2e). Johnsen’s score count further revealed that major spermatogenic defects in testis pieces of the busulfan group occurred around the stage of generating haploid germ cells (Fig. 2f,g)^32^. These data suggested that busulfan-mediated germ cell depletion in recipients affects the spermatogenesis of transplanted testis pieces.

### Germ cell depletion in recipient testes compromises the retinoic acid supply in donor testis tissue

**Figure 3:**
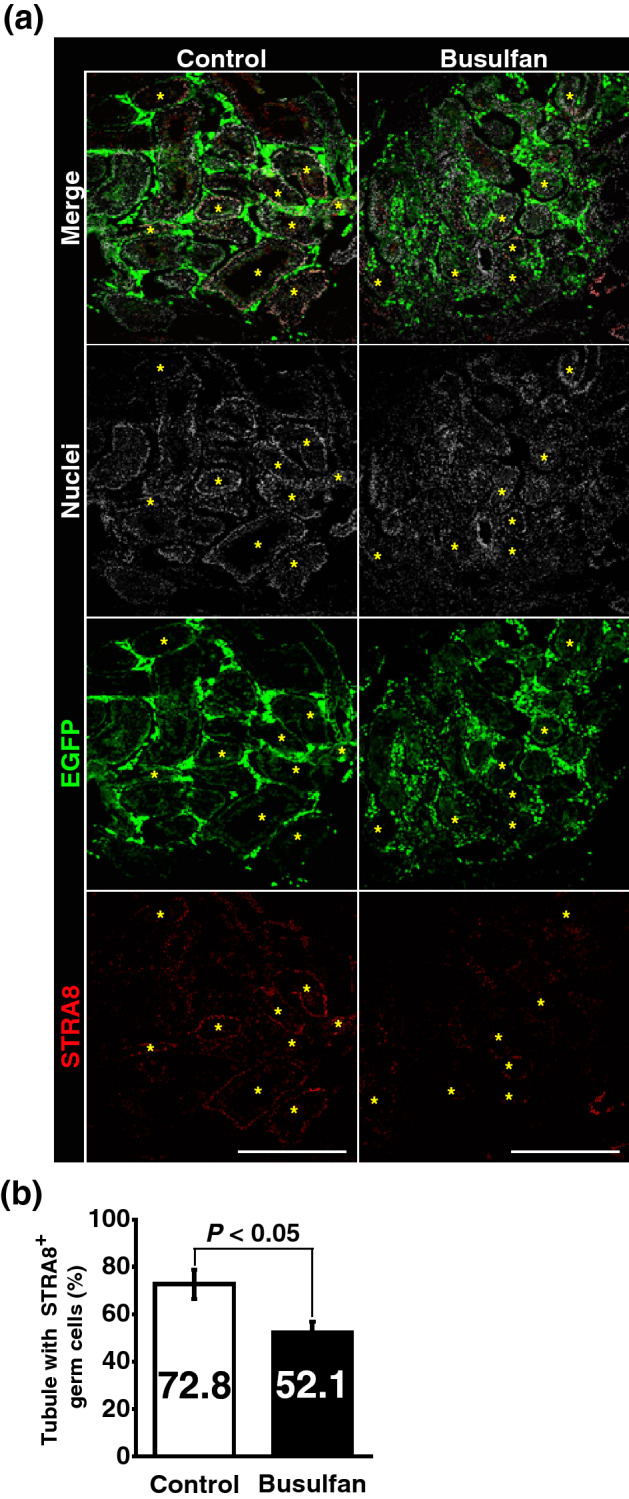
Recipient germ cell depletion compromises spermatogenesis in recipient testes transplanted into the testis interstitium. Testis pieces analysed in Fig. 2 were further analysed by immunofluorescence of STRA8. (**a**) STRA8 staining (red). EGFP^+^ transplanted tissues are green. Nuclei were counterstained with Hoechst 33,342 (grey). Asterisks (*) mark tubules carrying STRA8-expressing germ cells. (**b**) Quantitative analysis of tubules with STRA8-expressing germ cells in donor testis pieces of control and busulfan groups. Control group, n = 6 testes; busulfan group, n = 7 testes. A total of 2,507 tubules in which 725 tubules carried STRA8^+^ germ cells were analysed. Bar 500 μm (**a**).

To elucidate the mechanism of the spermatogenic defects in the transplanted testis pieces, we focused on niche functions. The germline niche allows SSCs to self-renew and differentiate to drive spermatogenesis by supplying soluble factors. GDNF is indispensable for the survival and self-renewal of SSCs, whereas RA induces SSC differentiation toward mature sperm via meiosis^1–3^. Testosterone produced by Leydig cells is also indispensable for the progression of meiosis^33^. It has been demonstrated that busulfan-treated testis produce testosterone^34,35^, whereas RA is synthesised by the combination of testicular cells including germ cells^36^. Based on this context, we next focused on RA signalling in transplanted testis tissues and found that the frequency of tubules containing STRA8^+^ differentiating spermatogonia/pre-leptotene spermatocytes was reduced significantly (Fig. 3a,b, SI Fig. S2). This observation suggests RA signalling defects in testis pieces transplanted into busulfan-treated testes.

**Figure 4:**
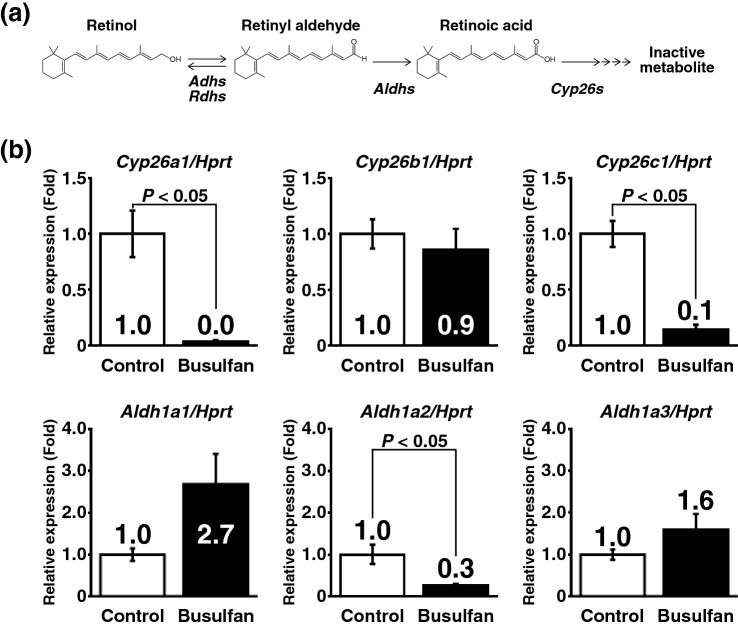
Expression of retinoic acid-related genes is compromised in germ cell-depleted testes. (**a**) Schematic representation of retinoic acid synthesis and metabolism in the mouse testis. Retinoic acid is synthesised from retinol via retinaldehyde. This process is mediated by ADHs, RDHs, and ALDHs. Synthesised retinoic acid contributes to regulation of spermatogenesis or is metabolised by CYP26 enzymes. (**b**) Quantitative RT-PCR analysis of testes with or without germ cell depletion induced by busulfan treatment. After normalisation to *Hprt* expression, values of the control group were set to 1.0 (n = 5 testes for control group, n = 6 testes for busulfan group).

Male germ cells and testicular somatic cells collaboratively synthesise *all trans*-RA by expressing enzymes that metabolise vitamin A/retinol^36^ (Fig. 4a). Quantitative reverse transcriptase-polymerase chain reaction (qRT-PCR) revealed that *Aldh1a2*, encoding the major RA-synthesising enzyme gene in the testis, was downregulated in busulfan-treated testes (Fig. 4b). Consistent with this finding, we also found downregulation of *Cyp26a1* and *Cyp26c1* genes (Fig. 4b). Our previous study revealed that RA induces these genes^37,38^. Therefore, the current data strongly suggest that germ cell depletion reduces testicular RA production.

### Retinoic acid administration ameliorates spermatogenic functions of testis pieces transplanted into busulfan-treated testes

Based on our observations, we hypothesised that RA administration ameliorates spermatogenic functions of testis pieces transplanted into busulfan-treated testes. To test our hypothesis, busulfan-treated recipients transplanted with testis pieces were administrated RA five times with an 8.5-day interval just prior to sacrifice (Fig. 1).

**Figure 5:**
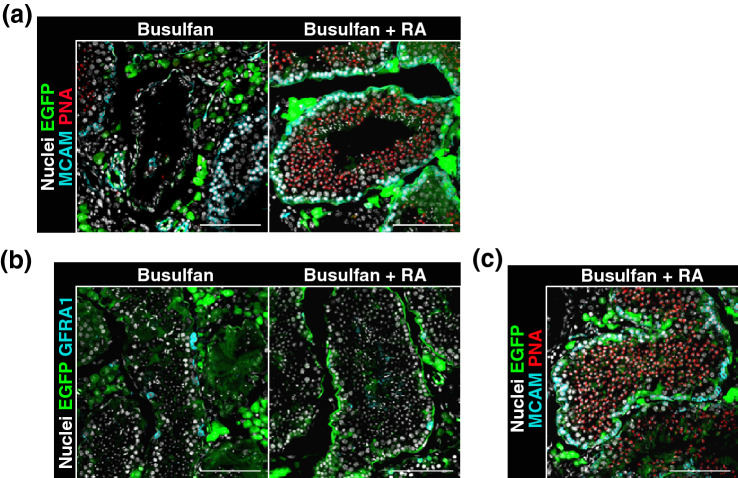
Repetitive RA treatment improves the spermatogenesis of testis pieces transplanted into the testis interstitium of germ cell-depleted mouse. At 153–154 days after testis piece transplantation, germ cell-depleted mice were sacrificed to collect transplanted tissues. In this experiment, mice in the busulfan + RA group were subjected to five RA treatments (750 μg/mouse/treatment) with an 8.5-day interval during the last 43 days. Collected tissues were analysed by immunofluorescence staining. (**a**) MCAM (cyan) and PNA (red) staining. Acrosomes were labelled with PNA. EGFP expression indicated that the tissues were derived from transplanted testis pieces. Nuclei were counterstained with Hoechst 33342 (grey). (**b**) GFRA1 staining (cyan). Nuclei were counterstained with Hoechst 33342 (grey). EGFP expression (green) indicated that the tissues were derived from donor testes. (**c**) Tubules with unusually arranged spermatogenic cells found in transplanted testis pieces of the busulfan + RA group. Note the excessive number of round spermatids and slight elongating spermatids, without a lumen in the tubule. Bar 100 μm (**a**–**c**).

As expected, RA administration improved the production of haploid germ cells (Fig. 5a, SI Fig. S3a). In the busulfan group (n = 7), 68.6 ± 4.3% of tubules contained PNA^+^ haploid germ cells, whereas these cells were found in 89.6 ± 1.3% of tubules in the busulfan + RA group (n = 4) (*P* < 0.01 by the Student’s t test; 4,917 tubules were analysed, among which 3,789 tubules carried PNA^+^ haploid germ cells). In addition, the population of MCAM^+^ cells was not affected. In the busulfan group (n = 7), 97.1 ± 1.4% of tubules contained MCAM^+^ haploid germ cells, whereas these cells were found in 99.6 ± 0.2% of tubules in the busulfan + RA group (n = 4) (no significant difference by the Student’s t test; 4,917 tubules were analysed, among which 4,838 tubules carried MCAM^+^ haploid germ cells). Concomitantly, Johnsen’s score count was also improved by this treatment [busulfan group, 6.4 ± 0.2 (n = 7); busulfan + RA group, 7.7 ± 0.1 (n = 4); *P* < 0.01 by the Student’s t test, 4,917 tubules were analysed].

However, we also found adverse effects. RA administration reduced the number of GFRA1^+^ undifferentiated spermatogonia in donor tubules (Fig. 5b, SI Fig. S3b). Tubules of the busulfan group carried 0.33 ± 0.02 GFRA1^+^ cells/tube (n = 7), whereas the busulfan + RA group carried 0.17 ± 0.04 cells/tube (n = 4) (*P* < 0.01 by the Student’s t test; 4,406 tubules carrying 1,192 GFRA1^+^ spermatogonia were analysed). In addition, tubules with an unusual germ cell arrangement were occasionally seen in the busulfan + RA group (Fig. 5c).

Taken together, these results support the idea that germ cell depletion in recipient testis results in RA deficiency, and demonstrate that RA administration augments reduced RA signalling in germ cell-depleted testes to recover spermatogenic activity. However, this treatment also exerts adverse effects on GFRA1^+^ spermatogonia and compromises the arrangement of germ cells in seminiferous tubules, suggesting an abnormality of haploid germ cells to produce the next generation of cells.

**Figure 6:**
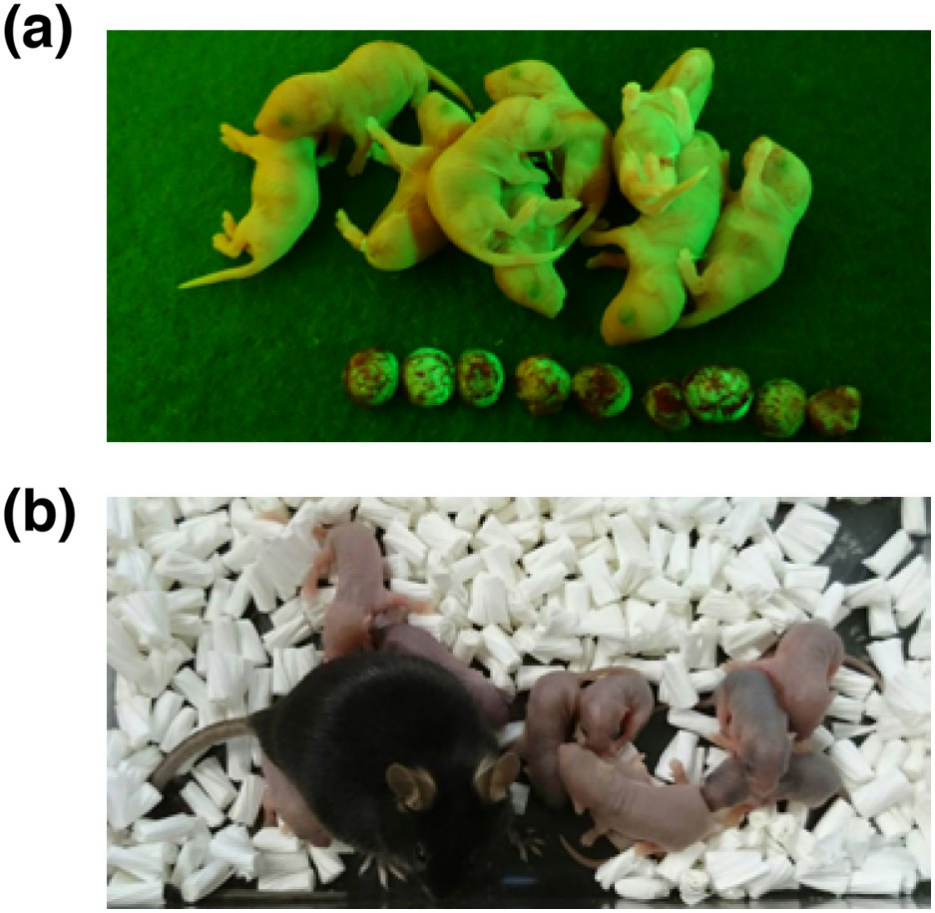
Retinoic acid treatment has no adverse effect on offspring. (**a**) Offspring were derived from EGFP^+^ haploid germ cells collected from transplanted testis pieces exposed to repeated retinoic acid treatments. (**b**) Offspring shown in (**a**) were fertile to produce healthy pups.

To determine whether healthy offspring could be produced from haploid germ cells generated in RA-administrated donor testes, we conducted in vitro microinsemination followed by transfer into oviducts of pseudo-pregnant females^39^. A transplanted testis piece was collected and immediately immersed in paraffin oil at 4 °C until germ cell preparation. Within 24 h, GFP^+^ haploid germ cells, including round and elongating spermatids, were collected under fluorescence stereomicroscopy and immediately subjected to intracytoplasmic sperm injection. As a result, of the 119 generated embryos, 90 (75.6%) developed to the two-cell stage within 24 h of culture. After transfer into the oviducts of four pseudo-pregnant females, 27 live pups were born (13 males and 14 females, Fig. 6a). These male and female offspring did not show any overt abnormalities and were fertile to produce a healthy next generation (Fig. 6b). These results indicate that RA treatment of the recipient does not exert an adverse effect on the next generation.

## Discussion

Busulfan-mediated germ cell depletion optimises testicular conditions for SSC transplantation^30,31^. Although this finding suggests that germ cell-depleted testes are also suitable for testis piece transplantation^27^, our present study revealed that germ cell depletion in recipient testes compromised spermatogenesis in orthotopically transplanted donor testis pieces at least in part by RA insufficiency. Indeed, repetitive supply of RA improves spermatogenesis in testis pieces transplanted into germ cell-depleted testes. Although there were some negative effects on GFRA^+^ spermatogonia, considering that RA-treated sperms produced healthy offspring that were also fertile, RA treatment was considered to be supportive for spermatogenesis.

Generally, recipient conditions affect the prognosis of transplantation. Shinohara and colleagues demonstrated that germ cell depletion releases the germline niche from occupation by resident SSCs^30^. In fact, the degree of germ cell depletion in recipient testes correlates positively with the frequency of SSC colonisation and subsequent germline transmission^31^. Considering the abundant GDNF in germ cell-free tubules and hyperproliferation of transplanted GFRA1^+^ spermatogonia in *Kit*^*W*^*/Kit*^*W-v*^ recipient tubules^40^, germ cell-depleted testes are likely to be suitable recipients for not only SSC transplantation, but also testis piece transplantation. Therefore, it was unexpected that germ cell depletion compromised the spermatogenesis of donor tissues.

Spermatogenesis is a complex process. Briefly, undifferentiated spermatogonia including SSCs produce differentiating spermatogonia that subsequently enter meiotic division after six mitotic divisions to produce haploid sperm^5^. In addition, various factors are known to contribute to this process. In particular, RA and testosterone are fundamental factors for the meiotic process. Indeed, disruption of RA synthesis or androgen production disrupts spermatogenesis^41–43^. In the present study, we focused on RA signalling, because germ cells contribute significantly to RA production by expressing *Aldh1a2* that is responsible for production of testicular RA. Quantitative RT-PCR confirmed that germ cell depletion resulted in RA insufficiency. Simultaneously, we found that expression of *Cyp26b1* was still maintained even after germ cell depletion. Of the three *Cyp26* genes, only *Cyp26b1* is indispensable for proper spermatogenesis^44^. Considering these observations, loss of *Aldh1a2* and maintained *Cyp26b1* expression in germ cell-depleted recipient testes should consume RA in orthotopically transplanted testis pieces. In fact, the frequency of tubules containing STRA8^+^ germ cells in donor testes was reduced significantly when recipient germ cells were depleted, and repetitive RA treatments rescued this effect to some extent. However, we also found that the present method often induced tubules with an unusual germ cell arrangement with a closed lumen. In this experiment, we employed repetitive RA administrations to compensate for the magnitude of RA signals. As described previously, periodical RA oscillation controls the seminiferous epithelial cycle with 8.6-day periodicity^2,3^. However, our protocol did not exactly follow the physiological RA oscillation. Although administrating the dose employed in the present study was sufficient to upregulate RA-inducible genes in testes with or without germ cells^37,38^, this dose might not be optimal to support proper spermatogenesis under the present experimental conditions. However, it should be considered that RA might cause adverse effects, especially on the undifferentiated spermatogonia population. Indeed, although Agrimson et al. reported that neonatal RA administration has no effect on the population of SSCs^45^, our RA administration protocol caused a reduction of GFRA1^+^ spermatogonia. These data suggest that testicular conditions and age affect the RA sensitivity of SSCs in the testis. Therefore, optimisation of the timing and dose of RA administration should improve spermatogenesis and SSC maintenance in transplanted testis pieces.

The present study did not investigate testosterone. Previous studies have demonstrated that busulfan treatment does not alter testicular testosterone^34,35^, whereas another report demonstrated that RA signal inhibition in Leydig cells results in abnormal spermatogenesis and epididymal functions^46^. The former two reports employed a single 30 mg/kg busulfan administration. However, this procedure might restore spermatogenesis from remnant SSCs^47^. This might also permit RA synthesis in the testis to some extent and lead to testosterone production. However, the present study used a higher dose (44 mg/kg) of busulfan at which germ cell-mediated RA synthesis is considered to be diminished completely. Such treatment might diminish the production of testosterone. Moreover, oestradiol, another sex steroid synthesized from testosterone by CYP19A/aromatase in Leydig cells and indispensable for proper spermatogenesis, might be affected by busulfan^13,14^. Considering these observations, RA insufficiency might also indirectly compromise spermatogenesis via modulated production of sex steroids in Leydig cells.

A GnRH analog supports colonisation of SSCs transplanted into infertile rodent testes by suppressing gonadotropin release from the pituitary grand^48,49^. In addition, repetitive administrations of the GnRH analog support recovery from busulfan damage for spermatogenesis^50^. These findings suggest that gonadotropin exerts adverse effects on the recovery process of spermatogenesis. In this context, busulfan treatment distinctly elevates LH and FSH levels in the pituitary and serum of rats^51^. Therefore, gonadotropin suppression in recipient might improve spermatogenesis in orthotopically transplanted donor testis pieces.

Altered expression of several factors might have also contributed to the reduced spermatogenesis in transplanted testis pieces. Our previous study demonstrated that GDNF suppresses *Rarg* in undifferentiated spermatogonia both in vitro and in vivo, and germ cell depletion upregulates testicular *Gdnf*^37,38^. Although the number of GFRA1^+^ spermatogonia was not changed in our present study, *Gdnf* upregulated in recipient testes might suppress *Rarg* expression in GFRA1^+^ spermatogonia of donor testes to suppress production of differentiating spermatogonia. Germ cell-derived PDGFA is also important for Leydig cell functions and proper spermatogenesis^52^. Restructuring of the molecular network by controlling these factors is beneficial for proper spermatogenesis in transplanted testis pieces.

RA causes various developmental defects in vertebrates because it plays fundamental roles in gastrulation, axis formation, and organ formation during development because of cell fate disruption^53^. In addition, RA alters the epigenome status of several cell types^54^. Moreover, alteration of the epigenome status in sperm results in developmental abnormalities of offspring^55^. However, the present study demonstrated that ectopic RA treatment did not cause any developmental defects in offspring.

The microinsemination technique was primarily developed by Yanagimachi and colleagues in 1995 using mice^39^. This technique has already been employed to assist human reproduction, and no overt abnormalities have been reported to date^16,17^. A combined technique including testis piece transplantation, RA treatment, and microinsemination might be practical to restore spermatogenesis, especially in prepubertal cancer patients.

Although it has been reported that recipient conditions affect the spermatogenesis of transplanted testis pieces^56^, most studies have employed subcutaneous transplantation, and other transplantation sites remain to be elucidated. The present study employed intratesticular transplantation and found that depletion of recipient germ cells compromised the RA production/metabolism ratio and reduced spermatogenesis of donor testis pieces transplanted into recipient testis. RA treatment supported the spermatogenesis of donor testis pieces when the size of the donor testis piece was insufficient for the size of the recipient testis after treatment with the anti-cancer drug. These findings may contribute to the treatment of prepubertal cancer patients to recover fertility after anti-cancer therapy. However, there are still many parameters (e.g. transplantation site, supportive molecules, and factors affecting prognosis) to be considered when applying testis piece transplantation to assisted reproductive technology.

## Methods

### Ethics statement

The institutional animal care and use committee of Shinshu University approved all animal experimentation protocols (Approval Nos. 260013, 280120, and 019032). All experiments were performed in precise accordance with the manual provided by the institutional animal care and use committee of Shinshu University.

### Testis piece transplantation and treatments

Male C57BL/6NCrSlc mice were purchased from Japan SLC (Shizuoka, Japan). Seven-week-old mice with/without treatment with 44 mg/kg body weight busulfan (Sigma-Aldrich, St Louis, MO, USA) at 3 weeks of age, which depletes germ cell in testes, were used as recipients. Male C57BL/6 Tg14(act-EGFP)OsbY01 ‘green’ mice were used as testis donors^57^. Testis pieces collected from newborn mice (1.5–4.5 days postpartum) were transplanted into recipient testes. Briefly, recipient mice were anesthetised with isoflurane (FUJIFILM Wako Pure Chemical Co., Ltd., Osaka, Japan) and a small cut was made in the tunica albuginea of each animal using fine forceps. A single testis piece was inserted into the testicular parenchyma^25^. In some cases, recipient testes were treated five times with *all-trans* retinoic acid (750 µg per mouse, Sigma-Aldrich) dissolved in a 10% ethanol-sesame oil (Nacalai Tesque, Kyoto, Japan) with 8.5-day intervals just prior to sacrifice (Fig. 1).

### Immunofluorescence staining

Recipient mice were sacrificed at around 146 days after transplantation, and donor recipient testes carrying donor testis pieces were collected. After overnight fixation in 4% paraformaldehyde/PBS (−) at 4 °C followed by overnight immersion in 30% sucrose/PBS (−), testes were embedded in Tissue-Tek OCT compound (Sakura Finetek, Tokyo, Japan) to prepare 8 µm-thick cryosections. After 10 min of treatment with 0.1% Triton-X 100 (Sigma-Aldrich) in PBS (−) at 4 °C followed by blocking with 10% donkey serum (Jackson ImmunoResearch Laboratories, Inc., West Grove, PA, USA), the sections were incubated with primary antibodies overnight at 4 °C. Sections were further incubation with secondary antibodies at room temperature for 1 h. Nuclei were counterstained with Hoechst 33342 (Sigma-Aldrich). Images were captured by confocal laser scanning microscopy (FV1000-D; Olympus, Tokyo, Japan) or All-in-one fluorescence microscopy (BZ-X710, KEYENCE Co., Ltd., Osaka, Japan). Collected images were joined to reconstruct an entire image of testicular cross-sections. Antibodies used in this study were: a rat anti-mouse CD146 monoclonal antibody (anti-mouse MCAM antibody, cat# 134702; clone ME-9F1; Biolegend Inc., San Diego, CA, USA); rhodamine-conjugated peanut agglutinin (RL-1072; Vector Laboratories, Burlingame, CA, USA); goat anti-rat GFRA1 antibody (AF560; R&D Systems, Inc., Minneapolis, MN, USA); rat anti-mouse GATA1 monoclonal antibody (sc-265; clone N6; Santa Cruz Biotechnology, Dallas, TX, USA); Alexa Fluor 647-conjugated donkey anti-rabbit IgG (A-31573; Thermo Fischer Scientific, Waltham, MA, USA); Alexa Fluor 647-conjugated donkey anti-rat IgG F(ab′)^2^ fragment (ab150151; Abcam, Cambridge, UK); rhodamine Red-X-conjugated anti-rat IgG (712-295-153; Jackson ImmunoResearch Laboratories, Inc.).

### Quantitative reverse transcriptase-polymerase chain reaction

Total RNA was prepared using Sepasol^®^-RNA I Super G (Nacalai Tesque). First-strand cDNA was synthesised using ReverTra Ace^®^ qPCR RT Master Mix with gDNA Remover (Toyobo Co., Ltd., Osaka, Japan). TB Green™ Premix Ex Taq™ II (Tli RNaseH Plus) (Takara Bio Inc., Shiga, Japan) in combination with a Thermal Cycler Dice^®^ Real Time System TP800 was used for quantitative PCR. Reaction conditions were as follows: 95 °C for 1 min, followed by 40 cycles of 95 °C for 15 s, 55 °C for 30 s, and 72 °C for 30 s, followed by a melting curve program. Each qRT-PCR was run in duplicate. Cycle threshold values were calculated by a second derivative maximum method using Thermal Cycler Dice^®^ Real Time System Software Version 5.10 (Takara Bio Inc.). The standard curve method was applied to determine the absolute quantity. A standard cDNA stock for absolute quantitation was prepared from the PCR products using a FastGene™ Gel/PCR Extraction Kit (Nippon Genetics, Tokyo, Japan). For data evaluation, values of the control group were set to 1.0 after normalisation to *Hprt* expression levels. Primer sequences are listed in Table 1.

**Table 1:** Primers for RT-qPCR.

*Gene*	Forward primer	Reverse primer
*Aldh1a1*	CTCCTGGCGTGGTAAACATT	CCATGGTGTGCAAACTCAAC
*Aldh1a2*	CCATGACTTCCAGCGAGAT	TCTGAGTTCTGCCATTCATTG
*Aldh1a3*	GTGGAGTTCGCCAAGAAGAG	AGAAGACCGTGGGTTTGATG
*Cyp26a1*	TGACCCGCAATCTCTTCTCT	GAGGAGCTCTGTTGACGATTG
*Cyp26b1*	TGGTCACTGGTTGCTACAGG	TGGGCAGGTAGCTCTCAAGT
*Cyp26c1*	CAAAATCCAGCAGGAGCTGT	AACCGTCCAGTTCAAAGGTG
*Hprt*	GCTGGTGAAAAGGACCTCT	CACAGGACTAGAACACCTGC

### Microinsemination

To assess the integrity of haploid germ cells in donor testes treated five times with retinoic acid injection, round and elongated spermatids were collected and subjected to intracellular sperm injection (ICSI)^39^. In brief, donor testes were recovered and transported overnight at 4 °C before ICSI using a previously published method for transportation of epididymides^58^.

Round and elongated spermatids were collected from donor testis pieces by simple repeated pipetting of seminiferous tubules and injected into oocytes of B6D2F1 mice (Japan SLC) using a Piezo-driven micropipetter (PrimeTech, Tokyo, Japan) as described previously^59^. Resultant zygotes were cultured for 24 h and then transferred into the oviducts of pseudopregnant female mice (ICR strain, Japan SLC). Offspring were delivered by caesarean section.

### Statistical analyses

Results are presented as means ± SEM. Significance of differences between means for single comparisons was determined by the Student’s t test. *P* < 0.05 was considered statistically significant.

## Supplementary information

Supplementary information

## Data Availability

There are no datasets for this manuscript to share via public repository.
