# Supplementary material for: Germ cell depletion in recipient testis has adverse effects on spermatogenesis in orthotopically transplanted testis pieces via retinoic acid insufficiency
Source: Sci Rep. 2020 Jul 1;10:10796. doi: 10.1038/s41598-020-67595-1 (PMC7330030; doi:10.1038/s41598-020-67595-1)
Supplement: Supplementary file 1 — Supplementary information [file 41598_2020_67595_MOESM1_ESM.pdf]

**Germ cell depletion in recipient testis has adverse effects on spermatogenesis in orthotopically transplanted testis pieces via retinoic acid insufficiency**

**Akihiro Tsuchimoto[1], Masaaki Tone[2], Narumi Ogonuki[3], Masashi Hada[3], Atsuo Ogura[3] and Seiji Takashima[1,2,4]**

[1]*Department of Textile Science and Technology, Interdisciplinary Graduate School of Science and Technology, Shinshu University, Ueda 386-8567, Japan*

[2]*Department of Applied Biology, Faculty of Textile Science and Technology, Shinshu University, Ueda 386-8567, Japan*

[3]*Bioresource Engineering Division, Bioresource Research Center, RIKEN, Tsukuba 305-0074, Japan*

[4]*Department of Biotechnology, Institute for Biomedical Sciences, Interdisciplinary Cluster for Cutting Edge Research, Shinshu University, Matsumoto 390-8621, Japan*

**This supplementary information contains three figures.**

(a)

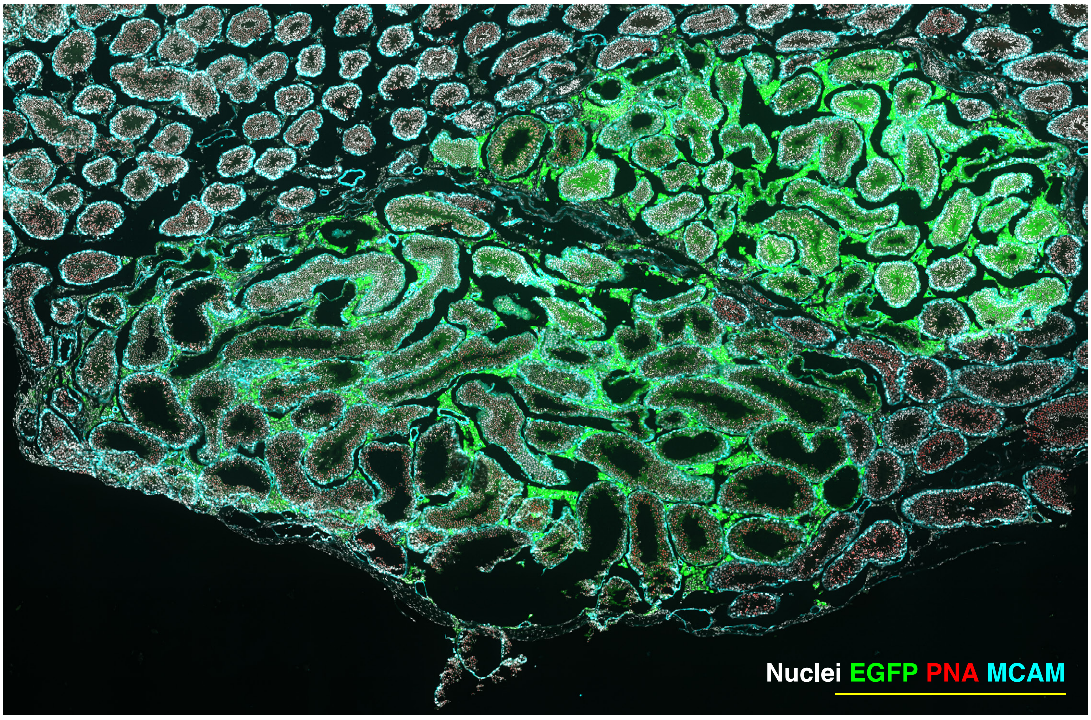


(b)

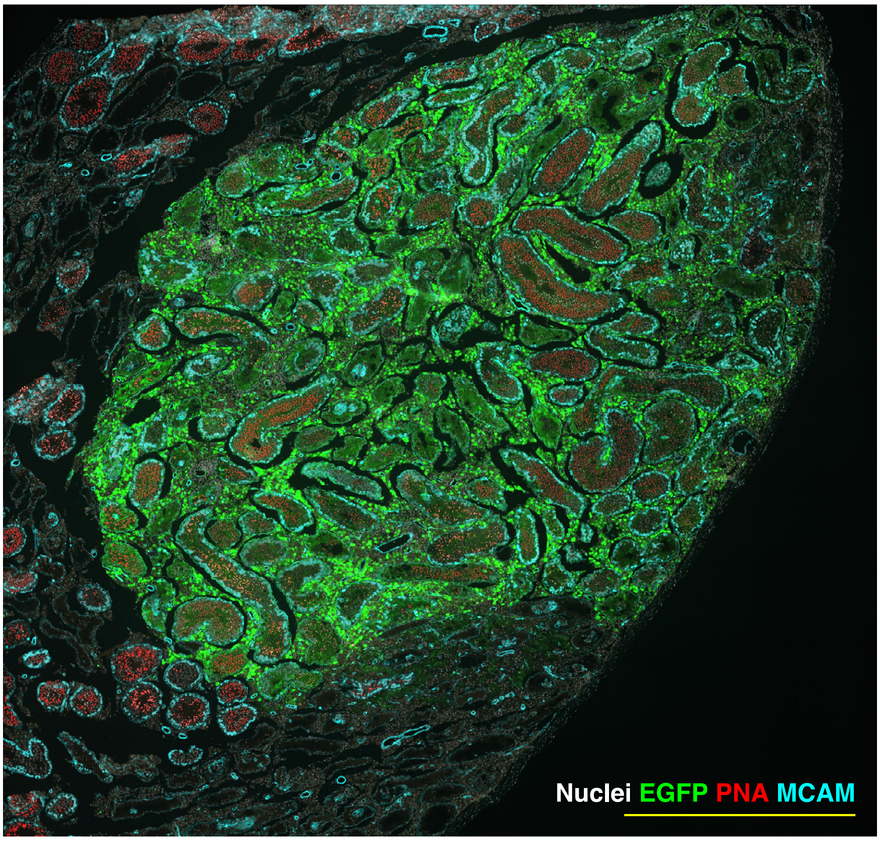

(c)

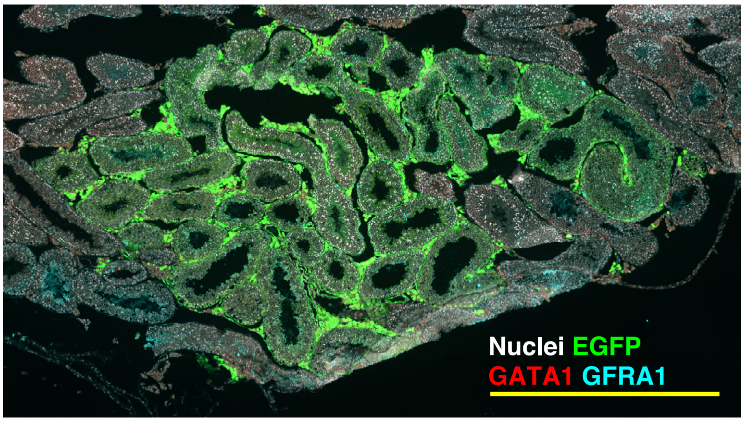


(d)

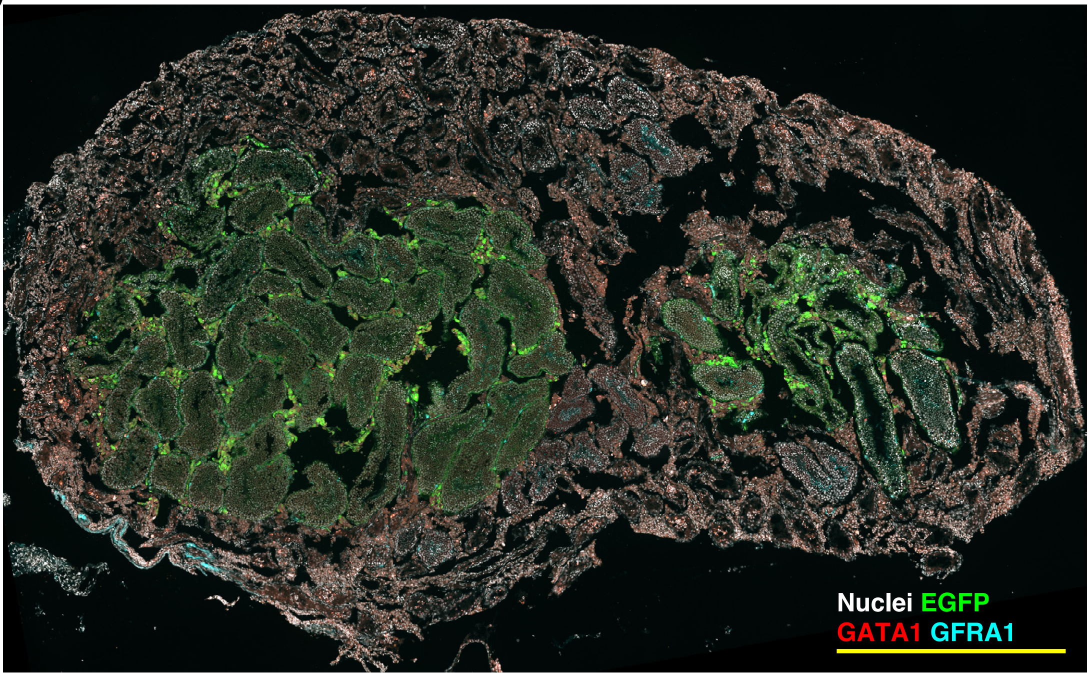


**Supplementary figure S1 (related to Fig. 2). Germ cell depletion in recipients compromises spermatogenesis in recipient testes transplanted into the testis interstitium.**

Representative images analysed in Fig. 2 are shown. EGFP (green) indicated that the tissues were derived from donor testes. (a,b) Immunofluorescence staining of MCAM (cyan) and PNA (red). (a) Testis from the control group. (b) Testis from the busulfan

group. (c and d) Immunofluorescence staining of GFRA1 (cyan) and GATA1 (red). (c) Control group. (d) Busulfan group. Although GATA1 is a Sertoli cell-specific marker and some Sertoli cell nuclei were stained in both control group and busulfan group, an obvious difference was not observed. Nuclei were counterstained with Hoechst 33342. Bar = 1 mm.

(a)

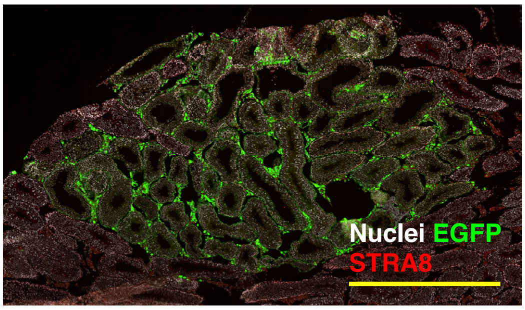


(b)

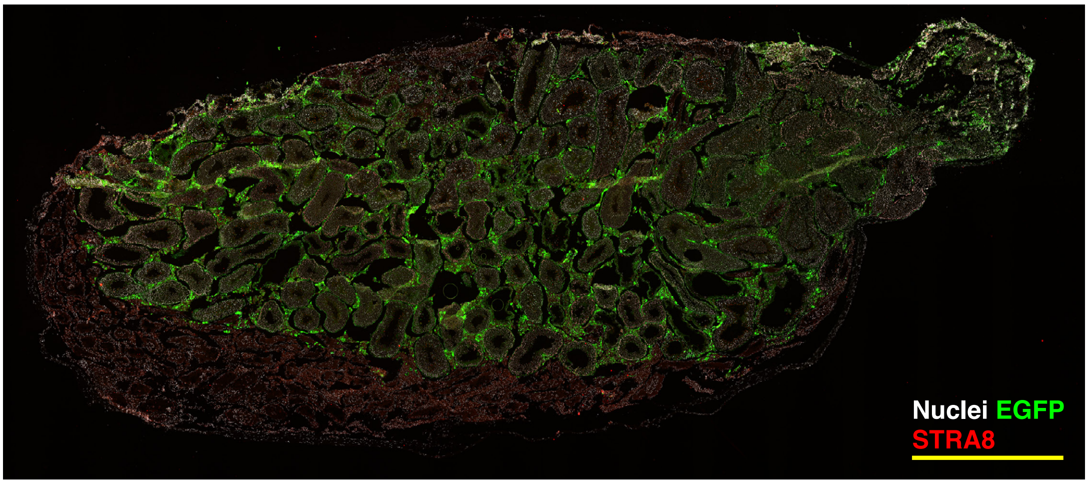


**Supplementary figure S2 (related to Fig. 3). RA signals in donor testes are attenuated in germ cell-depleted recipients by busulfan treatment.**

Representative images analysed in Fig. 3 are shown. STRA8 is red. Nuclei were counterstained with Hoechst 33342 (grey). EGFP (green) indicated that the tissues were derived from donor testes. (a) Testis from the control group. (b) Testis from the busulfan group. Bar = 1 mm.

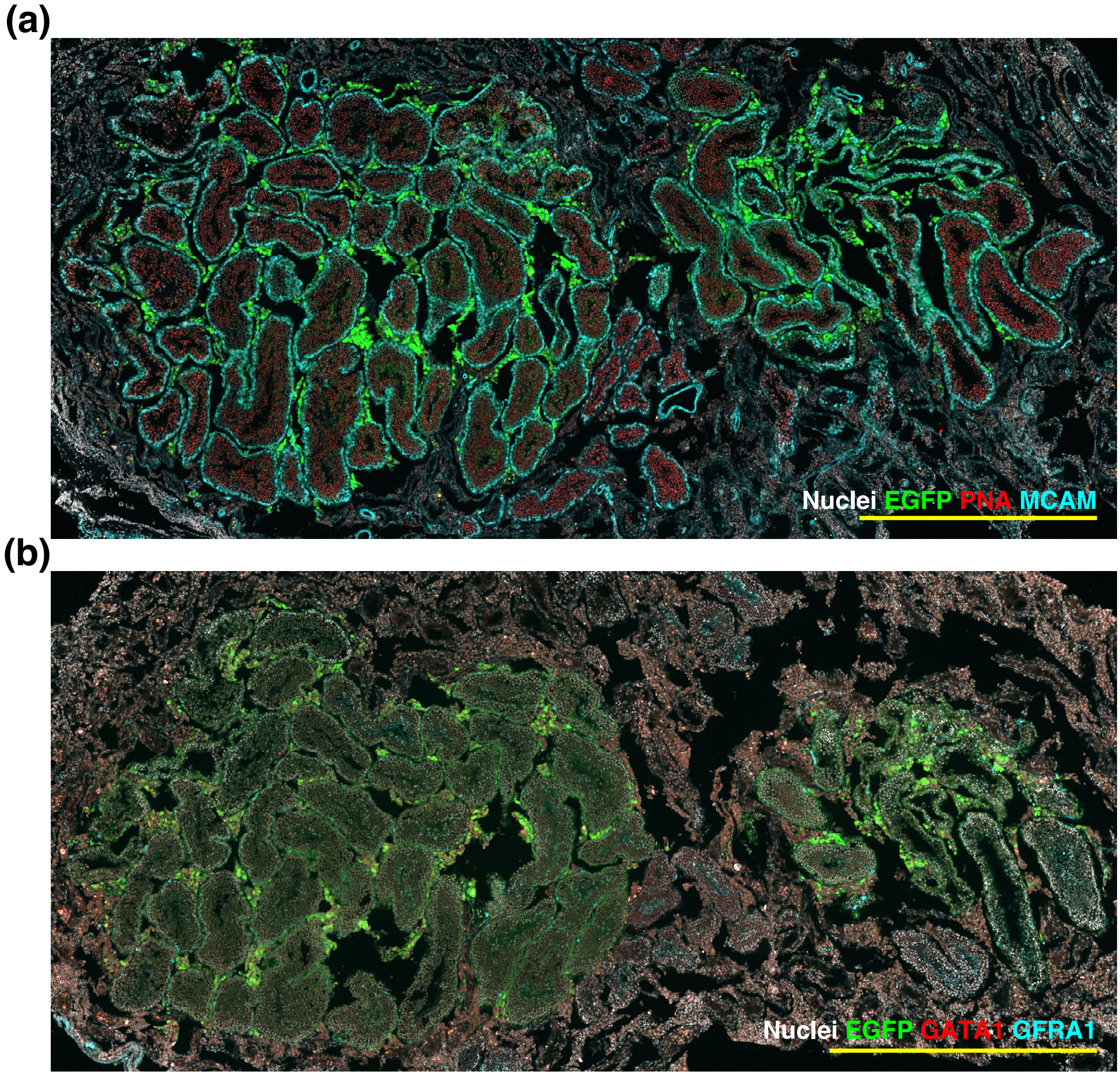


**Supplementary figure S3 (related to Fig. 5). Effect of RA treatment.**

Representative images analysed in Fig. 5 are shown. EGFP (green) indicated that the tissues were derived from donor testes. (a) Immunofluorescence staining of testis tissue from the busulfan + RA group. MCAM is cyan, and PNA is red. (b) Immunofluorescence staining of testis tissue from the busulfan + RA group. GFRA1 is cyan, and GATA1 is red. Although GATA1 is a Sertoli cell-specific marker and some Sertoli cell nuclei were stained in busulfan + RA group, an obvious change was not observed compared with the

busulfan group. Nuclei were counterstained with Hoechst 33342. Bar = 1 mm.
